# *Salmonella *serovars and their distribution in Nigerian commercial chicken layer farms

**DOI:** 10.1371/journal.pone.0173097

**Published:** 2017-03-09

**Authors:** Idowu Oluwabunmi Fagbamila, Lisa Barco, Marzia Mancin, Jacob Kwaga, Sati Samuel Ngulukun, Paola Zavagnin, Antonia Anna Lettini, Monica Lorenzetto, Paul Ayuba Abdu, Junaidu Kabir, Jarlath Umoh, Antonia Ricci, Maryam Muhammad

**Affiliations:** 1 Bacterial Research Division, National Veterinary Research Institute, Vom, Plateau State, Nigeria; 2 Reference Laboratory for *Salmonella*, Instituto Zooprofilattico Sperimentale delle Venezie, Legnaro, Padova, Italy; 3 Department of Veterinary Public Health and Preventive Medicine, Ahmadu Bello University, Zaria, Nigeria; 4 Department of Medicine, Faculty of Veterinary Medicine, Ahmadu Bello University, Zaria, Nigeria; Animal and Plant Health Agency, UNITED KINGDOM

## Abstract

Commercial poultry farms (n° 523), located in all the six regions of Nigeria were sampled with a view to generate baseline information about the distribution of *Salmonella* serovars in this country. Five different matrices (litter, dust, faeces, feed and water) were collected from each visited farm. *Salmonella* was isolated from at least one of the five matrices in 228 farms, with a farm prevalence of 43.6% (CI_95_[39.7–48.3%]). Altogether, 370 of 2615 samples collected (14.1%, CI_95_[12.8; 15.5%]) contained *Salmonella*. Considering the number of positive farms and the number of positive samples, it was evident that for the majority of the sampled farms, few samples were positive for *Salmonella*. With regard to the matrices, there was no difference in *Salmonella* prevalence among the five matrices considered. Of the 370 isolates serotyped, eighty-two different serotypes were identified and *Salmonella* Kentucky was identified as having the highest isolation rate in all the matrices sampled (16.2%), followed by *S*. Poona and *S*. Elisabethville. *S*. Kentucky was distributed across the country, whereas the other less frequent serovars had a more circumscribed diffusion. This is one of few comprehensive studies on the occurrence and distribution of *Salmonella* in commercial chicken layer farms from all the six regions of Nigeria. The relatively high prevalence rate documented in this study may be attributed to the generally poor infrastructure and low biosecurity measures in controlling stray animals, rodents and humans. Data collected could be valuable for instituting effective intervention strategies for *Salmonella* control in Nigeria and also in other developing countries with a similar poultry industry structure, with the final aim of reducing *Salmonella* spread in animals and ultimately in humans.

## Introduction

Nigeria is the largest market in sub-Saharan Africa with an estimated population of 174.5 million people in 2013 and a population growth rate estimated at three percent annually. The estimated gross domestic product (GDP) in 2012 was $450.5 billion, ranking Nigeria 31^st^ worldwide, with agriculture accounting for over 38% of non-oil foreign earnings and employing 70% of the labour force [1]. The poultry industry in Nigeria has been rapidly expanding in past years despite facing many problems such as avian influenza, the global financial crisis and inadequate credit [2]. The Nigerian poultry industry increased from 150,700 million chickens in 2005 to 192,313 million in 2010 [3]. Across the different regions of the country, the poultry sector is characterized by a low level of production and specialization and a general weak level of biosecurity[2].

In 2011, Nigerian hen egg production totaled 636,000 metric tonnes and was valued at $527.49 million, ranking 19^th^ in world hen egg production and the top producer in Africa [3]. Both large and small egg farms are scattered all over the country, although they are generally concentrated around the major urban centres [2]. Poultry meat and eggs are the major sources of animal protein in Nigeria, as in many developing countries, because of their affordability and acceptability [4, 5]. This source is, however, being threatened by diseases such as salmonellosis and avian influenza [1].

Salmonellosis is a bacterial disease affecting both humans and animals worldwide and Nigeria is not an exception. Although most of the infections in humans cause mild gastroenteritis, life-threatening systemic infections are common especially among high risk categories [6]. Invasive nontyphoidal *Salmonella* commonly cause infection among infants, children, elderly and immunocompromised individuals worldwide and especially in African countries, where these diseases are driven in part by co-infection with malaria or human immunodeficiency virus (HIV) [7]. Sources and modes of transmission of nontyphoidal *Salmonella* are still poorly understood in Africa due to the lack of coordinated national epidemiological surveillance systems [8].

In food-producing animals and especially in poultry, *Salmonella* is one of the leading causes of infection, and this has a direct impact on the global marketing of the respective food-producing animals and animal-derived food products [9]. Poultry salmonellosis related to host adapted serovars remains a major constraint on poultry production in all parts of Nigeria [10, 11, 12]. Farmers still experience great losses (due to mortality, morbidity and drop in egg production) caused by host adapted *Salmonella* serovars despite huge amounts spent on vaccination and medication. In early life, *S*. Pullorum causes extremely high mortality of both broilers and commercial laying birds. Similarly, older birds succumb heavily to other serovars of *Salmonella* and it is assumed that *Salmonella* infections of this category of birds are mainly due to *S*. Gallinarum [13]. In addition to these host adapted serovars causing systemic disease, poultry harbor in their gastrointestinal tracts zoonotic serovars with no apparent signs of illness. Hence, these *Salmonella* serovars can be present in feaces excreted by healthy animals and may be transferred to raw foods of animal origin through contamination during slaughtering and processing [14]. Generally, *Salmonella* in food producing animals, including poultry, manifests as long periods of latent carriage with occasional faecal shedding, which is the leading source of contamination of feed, water and environment [9]

Relatively few African countries report surveillance data on *Salmonella* and as such, very limited information is available on *Salmonella* isolation for this continent [15]. This is also the case in Nigeria, where the few studies conducted so far show different drawbacks, such as the limited number of samples considered, the lack of representativeness of the samples selected, lack of access to serotyping facilities, or the restricted geographical coverage. Raufu *et al*. (2013) [6], who collected samples from three poultry slaughterhouses and five intensively managed poultry farms in a circumscribed area of Nigeria, reported a *Salmonella* prevalence ranging from 2 to 16%. Moreover, a study conducted in three commercial hatcheries in the Jos area reported a prevalence of 9%, with *S*. Kentucky and *S*. Hadar as the most frequent serovars [16]. Finally, a study involving five farms in Awka reported a *Salmonella* prevalence of 38.3% in poultry droppings with *S*. Paratyphi A, *S*. Gallinarum and *S*. Typhimurium as the most common serovars [17].

The present study was, therefore, carried out to determine the prevalence of *Salmonella* and the circulating serotypes in a selection of Nigerian commercial chicken layer farms representative of the entire country, with the final aim of providing baseline data, which is the first step for developing an effective strategy for the control of salmonellosis.

## Materials and methods

### Sampling plan

A cross sectional study was carried out to determine the prevalence and the circulating *Salmonella* serovars in commercial chicken layer farms in Nigeria. Samples were collected from twelve states with the largest number of commercial poultry farms in Nigeria based on data obtained from Federal Department of Livestock and Veterinary Services (FDL&VS) on farm populations in the country. The study included two states from each of the six agro ecological zone based on their commercial poultry production. The selected states were Ogun, Imo, Edo, Lagos, Rivers, Enugu in the Southern part of Nigeria and Plateau, Kaduna, Kano, Katsina, Gombe and Bauchi in the North. Sample size of 475 farms was calculated using a prevalence of *Salmonella* of 50%, an allowable confidence level of 5% and a desired accuracy of 4.5% [18]. A slightly higher number of farms were selected by adding a 10% non-response rate to the calculated sample size. Therefore, a sample of 523 farms was included in the study according to a list of registered farms in the selected states provided by the FDL&VS and on the willingness of farmers to collaborate. The number of farms surveyed in each selected state and in each farm category was calculated proportionately to the total number of chicken layer farms in each state (Table 1). The sampling frame covered primarily farms with at least 500 laying hens. In states where the number of farms with at least 500 hens was smaller than the sample size calculated for that state, smaller farms were included as well. In that case, farms with more than 200 hens were preferably considered. Only one flock per farm was sampled and in cases of multiple age flocks, sampling focused on the oldest laying hens. Selection of farms took into account the risk that a small number of initially selected farms may not be sampled (e.g. because of early slaughtering), and therefore, a slightly higher number was selected.

**Table 1:** Number of samples collected, number of farms sampled and prevalence of *Salmonella* per state.

State	Number of farms as provided by the FDLVS	No. of farms sampled	No. of samples collected	No. of infected farms (%)	No. of positive samples (%)
Ogun	1363	110	550	72 (65.4)	135 (24.5)
Lagos	310	25	125	14 (56.0)	19 (15.2)
Edo	223	18	90	2 (11.1)	3 (3.3)
Rivers	273	22	110	11 (50.0)	13 (11.8)
Enugu	471	38	190	18 (47.4)	31 (16.3)
Imo	322	26	130	10 (38.5)	15 (11.5)
Gombe	136	11	55	3 (27.3)	3 (5.5)
Bauchi	260	21	105	12 (57.1)	19 (18.1)
Plateau	917	74	370	26 (35.1)	40 (10.8)
Kaduna	1351	109	545	33 (30.3)	55 (10.1)
Kano	558	45	225	21 (46.7)	31(13.8)
Katsina	297	24	120	6 (25.0)	6 (5.0)
**Total**	**6481**	**523**	**2615**	**228**	**370**
				**43.6% CI**_**95**_ **[39.7; 48.3]**	**14.1% IC**_**95**_ **[12.8; 15.5]**

### Farms characteristics

Poultry industries are located in different regions of Nigeria and there are differences in the producers’ profiles. Indigenous poultry (local chicken) are mostly raised in the north of the country by rural farmers on a subsistence level; grandparent stocks are generally brought from Europe and are concentrated in the south. Parent stocks and day-old-chicks are mostly produced in the south by big industries, while the eggs are produced everywhere, but mainly around the major urban centres by both big and small farms [1].

**Fig 1:**
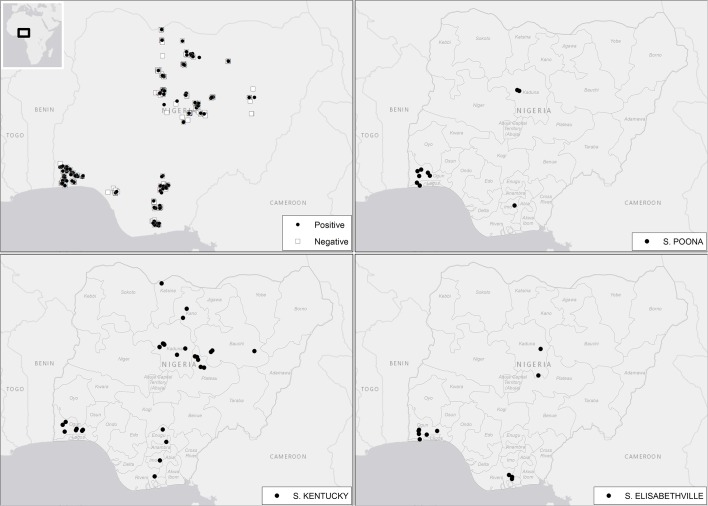
Distribution of *Salmonella* species across the twelve selected states in Nigeria. (A) Distribution of *Salmonella* spp. (B) Distribution of *S*. Poona (C) Distribution of *S*. Kentucky (D) Distribution of *S*. Elizabethville.

Geographical distribution of the sampled farms is shown in Fig 1.

The majority of farms (469) in this study were small flocks with between 200 and 10,000 birds, whereas large scale farms with over 10,000 birds were under-represented (54) since they are generally few in Nigeria.

In the southern part of the country, layers are often kept in cages, while in the north they are mainly kept on deep litters. Though, there are quite a number of poultry farms in the north, the capacity (flock size) is lower than what is obtainable in the south. This is as a result of the relatively hot climatic condition in the north limiting the opportunity of raising birds on a large scale.

Most small scale farms in Nigeria have generally poor infrastructure, poor sanitary and hygienic conditions and inadequate biosecurity measures in controlling stray animals, rodents, reptiles, amphibians. Disposal of poultry waste and by-products are usually on-farm thereby contaminating the environment. In some farms, faecal waste and litters are dried in sun and packaged close to poultry houses before selling to agro farmers as manure (fertilizer).

### Sample collection

Five different matrices (dust, litter, faeces, feed and water) were collected per each sampled farm. For each farm, one sample of each matrix was collected, making a total of 2615 samples. Dust samples (about 200 g per farm) were collected from different places in the farm house (walls, nest boxes and cages). Litter samples were collected using a commercially manufactured boot swabs (Technical Service Consultant, UK) worn on a sterile polyethene socking on a wellington boot (one boot swab per farm). Five fresh faecal droppings (about 200 g in total) were collected to make one sample. Approximately 200–300 g of feed was collected from different points on the farm. Sterile tongue depressors were used to collect dust, feed and faecal samples into sterile polyethene bags. Finally, for each farm, approximately 200 ml of unmedicated water was also collected.

All samples were immediately transported on ice to the nearest National Veterinary Research Institute (NVRI) outstation laboratory for storage at 4°C and subsequently transferred to NVRI Head Quarters in Vom within 24 hours.

The study was carried out on private farms and consent was sought from the farmers through the Federal Department of Livestock and Veterinary Services in the Ministry of Agriculture as well as all the avian influenza desk officers in each of the selected states. The study did not involve endangered or protected species.

### Culture and isolation

Faecal and environmental samples were pre-enriched in buffered peptone water (Oxoid, UK) in a 1:10 sample to broth ratio at 37°C for 18 hours. Mueller-Kaufmann tetrathionate (MKTTn) (Oxoid, UK) and Rappaport-Vassiliadis (RV) (Oxoid, UK) broths were both respectively inoculated with 1.0 ml and 0.1 ml of the pre-enriched broth. In addition, 100 μl (3 drops) of the pre-enrichment broths was inoculated onto Semi solid Rappaport-Vassiliadis (MSRV) Medium (Oxoid, UK) agar, and incubated at 42°C for 18 hours. Cultures from MKTTn, RV, and MSRV were plated simultaneously onto Brilliance *Salmonella* Agar (Oxoid, UK), Bismuth Sulphite Agar (Oxoid, UK) and Xylose Lysine Desoxycholate (Oxoid, UK) medium. The plates were incubated at 37°C for 18 hours (ISO 6579 Annex D) [19].

Typical *Salmonella* colonies were confirmed by biochemical assays including glucose fermentation, lactose oxidation, gas and hydrogen sulphide (H_2_S) production, the lack of β-galactosidase (ONPG) and lysine decarboxylation. *Salmonella* isolates were freeze-dried and shipped to the OIE *Salmonella* reference laboratory in Padova, Italy (Istituto Zoopofilattico Sperimentale delle Venezie) for serotyping according to the Kauffmann-White Scheme [20, 21]. Isolates, which were untypeable according to traditional serotyping, were tested by using the xMAP® *Salmonella* Serotyping Assay (Luminex, USA) to infer the serovar.

The matrices sampled as well as the analytical methods addressed the isolation of all *Salmonella* species including the zoonotic *Salmonella* serovars and were not only specifically intended for the identification of host adapted serovars such as *Salmonella* Gallinarum and Pullorum.

### Data collection and analysis

The data generated were entered into a Microsoft^®^ Excel 2007 data base. The frequency of each *Salmonella* serovar isolated as well as the frequency of *Salmonella* serovars from the different matrices was determined. Farm and matrix prevalence of *Salmonella* as well as the prevalence of *Salmonella* based on matrix in the twelve selected states of Nigeria with related 95% confidence intervals (CI_95_) were calculated. Cochran's Q test was used to verify if a significant difference existed among the *Salmonella* prevalence estimated for the different matrices, but also taking into account the dependence among samples originating from any one farm.

The statistical data analysis was generated using SAS software Version 9.3 (SAS Institute Inc. Cary, NC, USA).

The map (Fig 1) was generated using ESRI ® ArcMap^TM^ 10.0 –ArcGIS Desktop 10 (1999–2010 ESRI Inc.) The location and distribution of *Salmonella* positive farms were projected in the map using geographical coordinates of each farm.

The ArcGIS Online basemap “Light Gray Canvas” used in Fig 1 was compiled by Esri using HERE data, DeLorme basemap layers, Esri basemap data, and OpenStreetMap contributors (Esri, HERE, DeLorme, NGA, USGS).

## Results

A total of 228 out of the 523 farms sampled were positive for *Salmonella* with a farm prevalence of 43.6% (CI_95_[39.7–48.3%]). A farm was considered to be infected and/or contaminated when at least one matrix tested positive. Fig 1 shows the distribution of *Salmonella* positive farms in the selected states across the country. Looking at the prevalence of *Salmonella* per state (Table 1), the highest prevalence was recorded in Ogun state (65.4%), which was also one of the states with the largest number of farms sampled (110), whereas Edo state, which is among the states with the lowest number of farms sampled (18), registered the lowest prevalence (11.1%).

For each farm, one sample each of litter, faeces, dust, water and feed were collected and 370 out of the 2615 samples collected tested positive for *Salmonella* (14.1%, CI_95_[12.8; 15.5%]). Considering the number of positive farms and the number of positive samples (Table 1), it was evident that for each positive farm, more than one matrix was generally positive for *Salmonella*. Based on positive *Salmonella* farms, 155 farms had only one positive matrix, 50 farms had two positive matrices, 18 farms had three matrices, 4 farms had four matrices and only one farm had *Salmonella* isolated from all the five matrices. Table 2 provides details of the number and types of positive matrices per farm, whereas Table 3 provides details about the number of positive samples per matrix and state. Altogether, 52 farms had water contaminated with *Salmonella* (9.7% IC_95_ [7.3; 12.6]), whereas for the other matrices, the total numbers of positive matrices were similar and ranged from 63 for dust samples (12% IC_95_ [9.4; 15.1]) to 75 for faecal samples (14.3% IC_95_ [11.4; 17.6]). Cochran's Q test was not statistically significant (p = 0.1369), therefore, the probability of a sample being *Salmonella*-positive was similar for all five matrices considered.

**Table 2:** Number and type of positive matrices per farm.

No of positive matrices per farm	No of farms (%)	Details of the positive matrices
1	155 (68)	feed (34); litter (31); faeces (31); dust (30); water (29)
2	50 (21.9)	litter-dust (5); litter-faeces (8); litter-feed (5); litter-water (1); dust-faeces (9); dust-feed (4); dust-water (2); faeces-feed (9); faeces-water (3); feed-water (4)
3	18 (7.9)	litter-dust-faeces (2); litter-dust-feed (1); litter-dust-water (2); litter-faeces-feed (4); litter-faeces-water (1); litter-feed-water (3); dust-faeces-feed(1); dust-faeces-water (1); dust-feed-water (2); faeces-feed-water (1)
4	4 (1.8)	litter-dust-faeces-feed (2); litter-faeces-feed-water (1); dust-faeces-feed-water (1)
5	1 (0.4)	litter-dust-faeces-feed-water (1)

**Table 3:** Number of farms positive for *Salmonella* per each matrix in each selected state.

State (No. farm sampled)	N° of farms positive for *Salmonella*				
	Litter	Dust	Faeces	Feed	Water
Ogun (110)	24	19	31	25	20
Lagos (25)	4	0	5	4	4
Edo (18)	1	1	0	1	0
Rivers (22)	4	5	1	1	1
Enugu (38)	2	5	3	12	4
Imo (26)	1	5	2	4	2
Gombe (11)	0	0	2	1	0
Bauchi (21)	5	5	5	1	2
Plateau (74)	7	5	10	8	6
Kaduna (109)	13	11	12	9	3
Kano (45)	5	7	3	7	5
Katsina (24)	1	0	1	0	4
**Total (523)**	**67**	**63**	**75**	**73**	**52**
**Prevalence**	**12.8%**	**12%**	**14.3%**	**13.9%**	**9.7%**
**95% Confidence interval**	**10–16**	**9.4–15.1**	**11.4–17.6**	**11.1–17.2**	**7.3–12.6**

**Fig 2:**
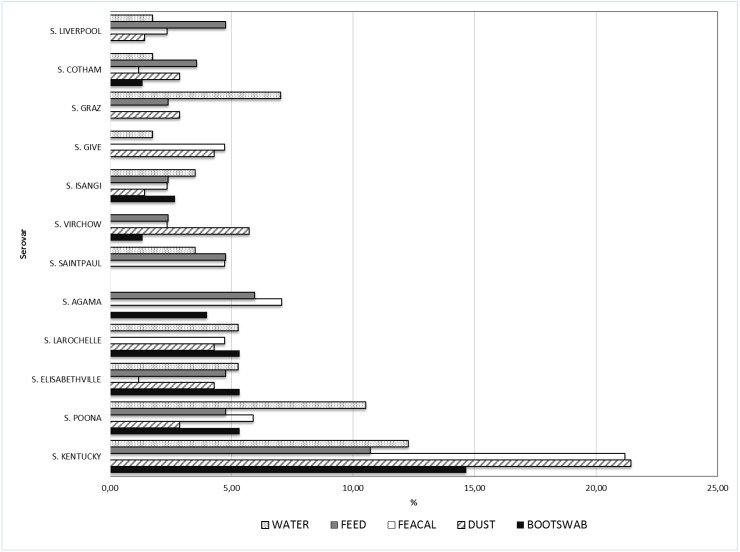
Relative frequency of selected *Salmonella* serovars isolated from the different matrices.

Of the 370 *Salmonella* isolates serotyped, 350 belonged to *S*. *enterica* subsp. *enterica*, one was *S*. *enterica* subsp. *diarizonae*, two were *Salmonella bongori*, while the remaining 17 isolates belonged to *S*. *enterica* subsp. *enterica*, but with an inconclusive serovar typing. A breakdown of the eighty-two (excluding the inconclusive *S*. *enterica* subsp. *enterica*) different serotypes identified from the five matrices showed *Salmonella* Kentucky was the most prevalent (found in 60 (16.2%) of samples) (Table 4). The other common serovars were *S*. Poona (21 isolates), *S*. Elisabethville (15 isolates), *S*. Larochelle and *S*. Agama (with 14 isolates each). Another serovar commonly associated with water was *S*. Graz (Fig 2).

**Table 4:** Species, subspecies and frequency of *Salmonella* serovars isolated.

S/N	*Salmonella* species	*Salmonella* subspecies	*Salmonella* serovars	Frequency of isolation (%)
1	*S*. *enterica*	*S*. *enterica* subspp. *enterica*	• *S*. Kentucky	60 (16.2)
			• *S*. Poona	21 (5.66)
			• *S*. *enterica*.subspp *enterica* (inconclusive)	17 (4.58)
			• *S*. Elisabethville	15 (4.07)
			• *S*. Larochelle • S. Agama	14 (3.77)
			• *S*. Saintpaul	10 (2.70)
			• *S*. Virchow • *S*. Isangi	9 (2.43)
			• *S*. Give • *S*. Graz • *S*. Cotham • *S*. Liverpool	8 (2.16)
			• *S*. Muenster • *S*. Telelkebir • *S*. Nigeria • *S*. Jangwani • *S*. Kingston	7 (1.89)
			• *S*. Hadar • *S*. Weltevreden • *S*. Berlin	6 (1.62)
			• *S*. Typhimurium • *S*. Corvallis • *S*. Canada • *S*. Dugbe	5 (1.35)
			• *S*. Goldcoast • *S*. Rubislaw • *S*. Chomedey	4 (1.08)
			• *S*. Namoda • *S*. Ridge • *S*. Vinohrady • *S*. Sanktmarx • *S*. Gaminara • *S*. Yaba • *S*. Carno • *S*. Kibi • *S*. Johannesburg • *S*.Schwarzengrund	3 (0.81)
			• *S*. Offa • *S*. Agbeni • *S*. Halle • *S*. Durham • *S*. Hato • *S*. Alachua • *S*. Plymouth • *S*. Chichester • *S*. Madjorio	2 (0.54)
			• *S*. Bonariensis • *S*. Fresno • *S*. Benfica • *S*. Lattenkamp • *S*. Stanleyville • *S*. Lexington • *S*. Be • *S*. Deversoir • *S*. Enteritidis • *S*. Livingstone • *S*. Lomita • *S*. Gege • *S*. Bradford • *S*. Mapo • *S*. Ealing • *S*. Mbandaka • *S*. Miami • *S*. Sangera • *S*. Millesi • *S*. Kisarawe • *S*. Kande • *S*. Sculcoates • *S*. Herston • *S*. Adelaide • *S*. Dumfries • *S*. Urbana • *S*. Nieukerk • *S*. Kuessel • *S*. Amba • *S*. Dallgow • *S*. Amina • *S*. Muenchen • *S*. Bedford	1 (0.27)
		*S*. *enterica* subspp. *diarizonae*	• *S*. IIIb 8: r: z	1 (0.27)
2.	*S*. *bongori*		• *S*. V 6,14: e,n,z15: - • *S*. V 48: i:-	2 (0.54)

In the majority (68%) of the positive farms, all isolates obtained from the same farm belonged to a single serovar. In 23.7% of the positive farms, two different serovars were found, while 8.3% of the positive farms had three or more serovars identified. Moreover, two different farms had five/six different serovars identified among their isolates. The distribution of the most common serovars (*S*. Kentucky, *S*. Poona and *S*. Elisabethville) across the country was analysed (Fig 1). It was evident that *S*. Kentucky, with the highest prevalence, was also widely distributed throughout all the states except Edo state, whereas *S*. Poona and *S*. Elisabethville showed a more circumscribed diffusion as they were identified in farms located far from each other. The distribution of *Salmonella* serovars among different matrices is shown in Fig 2.

## Discussion

This is the first comprehensive study on the occurrence and distribution of *Salmonella* in commercial chicken layer farms from all the six regions of Nigeria. A relatively high farm prevalence (43.6%) of *Salmonella* was detected among commercial poultry farms in Nigeria with state prevalences ranging from 11.1 to 65.4%. The prevalence reported in the present study is far above what was described for laying hens in EU countries, which have an overall prevalence of zoonotic *Salmonella* serovars of 2.5%, thanks to the successful control programs implemented in their poultry farms during recent years [22]. In other developed countries like the US, different situations have been described with contamination rates of up to 60% [23]. In Japan, *Salmonella* was isolated from 36% of broiler faecal samples [24]. Considering studies conducted in countries with a poultry industry structure similar to that described in the present study, Dione *et al*. (2011) [25] reported that in Gambia, the prevalence of *Salmonella* in chicken faeces was higher (67%) than in the current study. Another small-scale study conducted in the province of N'Djamena, Chad, described a prevalence for *Salmonella* in laying hen flocks of 42% [26], similar to the situation described by Andoh *et al*. (2016) [27] for laying hen and broiler farms in Ghana. Finally, looking at the Asian situation, Barua *et al*. (2012) [28] reported 18% prevalence in chicken layer farms in Bangladesh, whereas Lettini *et al*. (2015) [29] determined a prevalence of 46.3% from breeder, broiler and laying hen farms in Central Vietnam. These differences in isolation rates could be due to differences in terms of *Salmonella* status among countries, but could be influenced also by a plethora of different aspects such as sample types, collection seasons, culture methods, laying period, isolation methodologies, culture media, housing system and local environmental conditions [30].

Among the matrices, the highest *Salmonella* prevalences were in faeces (23%) and feed (22.7%) matrices and closely followed by litter (20.3%) and dust (18.9%) matrices. Water samples had the lowest prevalence of 15.1%. The prevalence of *Salmonella* was non-significantly higher in faecal samples than in other matrices. This result does not agree with the work of Tabo *et al*. (2013) [26], who reported the highest prevalence of 15.63% in litters rather than in faecal samples. The high proportion of positive dust samples we found suggests that once the environment is contaminated with *Salmonella*, the pathogen persists within the poultry flocks. Moreover, the high rate of *Salmonella* isolation from feed and water samples could be an indication of poor sanitation, handling and contamination along the poultry production chain as well as cross contamination, which calls for serious concerns. The high prevalence of *Salmonella* in water (although water was the least contaminated of the five matrices we studied) is of particular concern since every time the birds drink the water they are exposed to its microbial load. Water can be contaminated in two major ways; a) the water supply itself, especially when the water source is from well water or surface water, or b) bacterial contamination of water supply as a result of poor hygiene management [31].

In Nigeria, poultry feed is considered the most expensive component of poultry production. The feed is usually compounded using blood meal, bone meal, fish meal, egg shells (animal), groundnut cake and soya bean cake (plant) as sources of protein and calcium. In most cases, these ingredients are not properly preserved or un-hygienically packaged, thus serving as a source of contamination in feed. The climatic weather in Nigeria is warm and humid and *Salmonella* organisms can, under these circumstances, multiply in the feed especially during farm storage and administration [32]. Contamination may also occur during processing, transport and distribution of compounded feed mixture as most farmers, in a bid to save cost, would either compound their own feed (on the same farm with the birds) or purchase from a local un-hygienic feed mills. This high variety of ingredients used to produce poultry feed as well as the high level of diversification among the farms in the feed production and processing and the general low level of hygienic practices can explain the high prevalence of *Salmonella* in feed samples and the heterogeneity of serovars isolated from this source.

Further investigations could be useful to clarify the sources of infections and factors leading to the widespread isolation of *Salmonella* in Nigerian laying hen farms. Nevertheless, the high prevalence rate found in all the sampled matrices is in agreement with the primitive level of the infrastructure and bio-security measures observed on some of the farms involved in the study. Data collected demonstrated that there is an urgent need to improve personnel awareness about the importance of implementing good practices and sanitary measures in order to curtail *Salmonella* infections in Nigerian poultry farms. Aside from resource constraints, several measures are suggested to limit vertical and horizontal transmissions of *Salmonella* on farms and make the birds less vulnerable to *Salmonella* [27, 33, 34]. Namely, to ensure *Salmonella*-free feed and water, implementing effective cleaning and disinfection of the farms, applying appropriate measures against animate and inanimate vectors, and improving the sanitary status of animals should be implemented.

Environmental sampling of poultry houses is regarded as a cost effective method for the isolation of *Salmonella* from a large number of other competing bacteria [35, 36]. The samples generally collected from poultry houses to monitor the *Salmonella* status of a poultry flock include faeces, litter and dust [37]. Naturally, pooled faeces and dust sampling is considered more effective than individual faeces for detection of *Salmonella* in laying flocks [38]. While fresh faeces provide an indication of current infection, dust samples may be more indicative of previous infections, since *Salmonella* has been reported to survive in dust for up to 53 weeks [36].

In contrast to European data, which described twice the likelihood of *Salmonella* isolation from dust than faecal samples [39], we detected relatively similar prevalences in the different sample matrices (dust, litter and faeces). Our findings confirmed the results described by Andoh et al. (2016) [27], who conducted a cross-sectional study of poultry flocks in Ghana and suggested that the reduced detection rates in dust compared to faeces could be due to relatively high temperatures in dusts, a consequence of the climatic conditions. Moreover, sampling strategies that are appropriate to detect positive flocks in the European setting, with a negligible number of positive flocks, may not be effective in high prevalence situations, as in Nigerian chicken layer farms, where a relatively similar prevalence of *Salmonella* infection was revealed for all the matrices sampled.

The occurrence of *Salmonella* serotypes differed slightly among sample types and states investigated. However, most of the serovars were isolated from all the sample matrices. Serogroups B, C1 and C2-C3 belonging to subspecies I were dominant. This may be due to the ubiquitous nature of serovars in this subspecies and the result was consistent with results from the USA [40]. In Nigeria routine vaccination for fowl typhoid (*Salmonella* Gallinarum) is practiced by most farmers due to the availability of a locally produced *Salmonella* Gallinarum 9R strain vaccine which could induce cross-protective immunity against *Salmonella* of the same serogroup. Thus, the inability of this study to isolate the host adapted *Salmonella* serovars was a welcome development and calls for further investigation. However, even though the *Salmonella* Gallinarum 9R vaccine may be effective, there is no cross-protection between serotypes belonging to different serogroups. Vaccination against other serotypes of *Salmonella* is generally not practiced in Nigeria even though it is known that the immune response of poultry to vaccination could reduce the duration and severity of *Salmonella* infections and help prevent reinfection [41]. Therefore, if control of salmonellosis in Nigeria is to be embarked upon by vaccination, it should involve a multivalent vaccine targeting members of these serogroups.

The most predominant serotypes were *S*. Kentucky (16.2%), *S*. Poona (5.66%), *S*. Elizabethville (4.04%) and *S*. Larochelle/*S*. Agama (3.77%). While *S*. Kentucky had a widespread distribution in eleven out of the twelve states sampled, others were not as widespread as this serovar. Previous studies carried out in Nigeria also reported *S*. Kentucky as one of the most prevalent *Salmonella* serovars in poultry and in humans [6, 42, 43, 44]. This serovar, which was initially thought to be endemic in poultry industries in Africa, is rapidly spreading across different continents of the world and increasingly becoming a public health concern [45, 46]. The other serovars commonly isolated from Nigerian chicken laying hen farms are rarely described in literature with the exception of *S*. Poona, which was identified in multiple *Salmonella* outbreaks associated with pet turtles [47, 48] and linked to the consumption of contaminated cantaloupe [49].

Intriguingly, *S*. Enteritidis and *S*. Typhimurium, which are the most common agents of foodborne disease worldwide and the serovars most frequently involved in salmonellosis outbreaks [50], seem to have a marginal role in the epidemiology of *Salmonella* in Nigerian laying hen flocks, according to the data collected in the present study.

In Nigeria, as for the rest of Africa, it is very difficult to have a clear picture about the real situation of human salmonellosis due to the non-availability of facilities to provide essential tests for the diagnosis of *Salmonella* infections [51]. However, the limited amount of study concerning non-typhoidal *Salmonella* serovars responsible for human infections in Africa reported *S*. Enteritidis and *S*. Typhimurium as the most prevalent serovars [51]. Moreover, a recent study monitoring *Salmonella* from different sources, including humans, in the north-eastern regions of Nigeria reported *S*. Eko, *S*. Enteritidis and *S*. Hadar as the most common serovars from humans [6], whereas these serovars did not enter among the most common serovars found in chicken layer farms in Nigeria according to the data collected.

In a report published by FAO [1] it was reported that grandparent stocks are generally imported from Europe, but the lack of regulations and strict enforcement of laws on the importation of uncertified poultry and poultry products may be a problem in Nigeria. It remains to be ascertained to what extent the high *Salmonella* prevalence observed in this study was due to the introduction of infected birds from other countries or due to the infection of the animals once they were farmed in Nigeria. The high prevalence and presence of multiple *Salmonella* serovars throughout the country may be due to poor sanitary conditions of poultry farms, frequent movement of people and lack of enforcement of monitoring programmes especially for imported animals as well as the poorly managed borders with neighboring countries. Improving these conditions together with improved cleaning and disinfection could have a significant impact on reducing *Salmonella* levels on farms in Nigeria. Although vaccination is still regarded as an important part of the overall control strategy for *Salmonella*, it is, however, advocated that routine vaccination for *Salmonella* control should not stop at fowl typhoid control alone, but should include other serotypes which could be easily transmitted in eggs and poultry meat meant for human consumption. The circulation of zoonotic *Salmonella* in Nigeria, as in other developing countries, may have a global impact in terms of public health because of movements beyond the area of origin, thanks to trade and travel [28]. Knowledge about the extent of the phenomenon is essential in order to find feasible control measures at global level. Moreover, comparison of livestock and human isolates could discern the possible contribution of different sources to the burden of human salmonellosis.

In conclusion, this study collected baseline data on the prevalence and serotype distribution of *Salmonella* in Nigerian commercial poultry farms. The relatively high prevalence rate could be linked to general poor infrastructure and low biosecurity measures of chicken layer farms in the country and can form the basis for instituting effective intervention strategies.
